# Highly divergent karyotypes and barcoding of the East African genus *Gonatoxia* Karsch (Orthoptera: Phaneropterinae)

**DOI:** 10.1038/s41598-021-02110-8

**Published:** 2021-11-23

**Authors:** Elżbieta Warchałowska-Śliwa, Beata Grzywacz, Maciej Kociński, Anna Maryańska-Nadachowska, Klaus-Gerhard Heller, Claudia Hemp

**Affiliations:** 1 Institute of Systematics and Evolution of Animals, Polish Academy of Sciences, Sławkowska 17, 31-016, Kraków, Poland; 2 Magdeburg, Germany; 3 Department of Plant Systematics, University of Bayreuth, Bayreuth, Germany

**Keywords:** Cytogenetics, Evolutionary biology, Chromosomes, Entomology, Phylogenetics, Speciation, Taxonomy

## Abstract

East Africa is a hotspot of biodiversity of many orthopteran taxa, including bushcrickets. *Gonatoxia* Karsch, 1889 species are fully alate Phaneropterinae, which are perfectly adapted to the foliage of forests. We examined five species using combined cytogenetic and molecular data to determine the inter- and intraspecific genetic diversity. The variation in the diploid number of chromosomes in males ranged from 2n = 28 + X0 and 26 + X0 to 2n = 6 + X0. Fluorescence in situ hybridization showed from one to many 18S rDNA loci as well as interstitial sequences, especially in *G. helleri*. 18S rDNA loci coincided with active NOR and C-banding patterns. The isolation of populations of the species explains differences in the number of chromosomes (*G. maculata*), chromosomal polymorphism and chromosomal heterozygosity (*G. helleri*). Our molecular phylogeny based on the COI locus supported the monophyly of the genus *Gonatoxia* and separateness of the five examined species in accordance with their morphological features and chromosome numbers as well as the species’ distribution.

## Introduction

Differences in chromosome number (karyotype variability) or chromosomal polymorphism within species are seen in many plant and animal groups, and may be involved in adaptation e.g.^1–3^. In closely related taxa that differ in many structural features of their chromosomes, either numerically or morphologically, these changes may reveal reproductive isolation^4^. Variation in chromosome number may indicate that a genus/species underwent speciation processes concomitantly with chromosomal rearrangements (CRs), which play a role in speciation via reproductive isolation^5^. Recent theoretical and empirical studies have suggested that CRs can alter the rate and pattern of gene flow within or between species, through a reduction in the fitness of chromosomal hybrids, or by reducing recombination rates in rearranged areas of the genome, according to the suppressed-recombination model of chromosome speciation^6–9^. CRs have been shown to play a causative role in isolating species or populations in various insect genera^1,10–16^ including Orthoptera e.g.^17–24^. An interesting example of the association CRs with evolution and adaptation to changing environments is *Trimerotropis*. This American genus is characterised by a high incidence of inversions and inversion polymorphisms, and exhibit a clinal distribution of inversions polymorphism and evolutionary significance of inversion clines^21–24^.

The analysis of karyotypes is a useful tool for systematic and evolutionary studies because closely related species tend to have more similar karyotypes than more distinctly related ones^25^, although this is not always the case. Extreme cases of rapid chromosome evolution are found in insects e.g.^10,26–28^.

Different banding techniques are the most frequently used methods to identify the presence of structural rearrangements that contribute to speciation^7^. Fluorescence in situ hybridization (FISH) paired with classical cytogenetics, provides more detailed comparisons between the karyotypes of different taxa, and accurately identify CRs between species and/or genera. The number and location of rDNA signals (FISH) with distribution of heterochromatin (conventional chromosome banding) are useful to study karyotype evolution, identification of genus- and species-specific patterns as well as describing and delimiting new taxa within tettigoniid bushcrickets (e.g.^29–32^).

To better understand the role of chromosomal changes in species diversification, a correlation between chromosomal and genetic variability needs to be examined in the context of species’ geographical distributions, especially comparing endemic with widespread species (e.g.^11,17,33–36^).

Mountainous East Africa is known for its high degree of biodiversity and endemism, especially along the so-called Eastern Arc Mountains, stretching through Tanzania and southern Kenya. East Africa is a hotspot of biodiversity of many orthopteran taxa, including bushcrickets^37–39^. In the last few years, numerous papers have been published on East African Phaneropterinae taxa, including descriptions of several new genera and many species combined with genetic studies, mostly at the chromosome level e.g.^40–49^. Various studies indicate that during climatic fluctuations during the Plio-Pleistocene, habitats were ecologically fragmented, resulting in genetic isolation and speciation in mountainous areas and savanna forests in East Africa e.g.^50,51^. This region is an ideal natural laboratory for testing species boundaries and gene flow between different morphological forms, studying the influence of climatic factors on speciation and their mechanisms, and determining time scales of diversification of different groups e.g.^50–55^. Several hypotheses including refugial and geographical uplift models have been developed to explain distribution patterns and speciation mechanisms in lowland forests, as well as in montane regions of Central and Eastern Africa. Some studies^56–58^ provided evidence for the relevance of the fragmentation of a once continuous East African habitat as a major driver for the evolution of the high diversity found in this topographically rich landscape. Rapid speciation was not only observed in flightless species with low mobility (e.g. in Lentulidae^55^, and Karniellina^51–53,57,59–61^ or Hexacentrinae^39^), but also in fully alate and thus mobile taxa (e.g. in *Eurycorypha*^37^ or *Amytta*^62^). Orthoptera were also first forest dwellers and later adapted to open land habitats in Africa due to the aridification of Africa beginning from 8 mya^51^.

**Figure 1:**
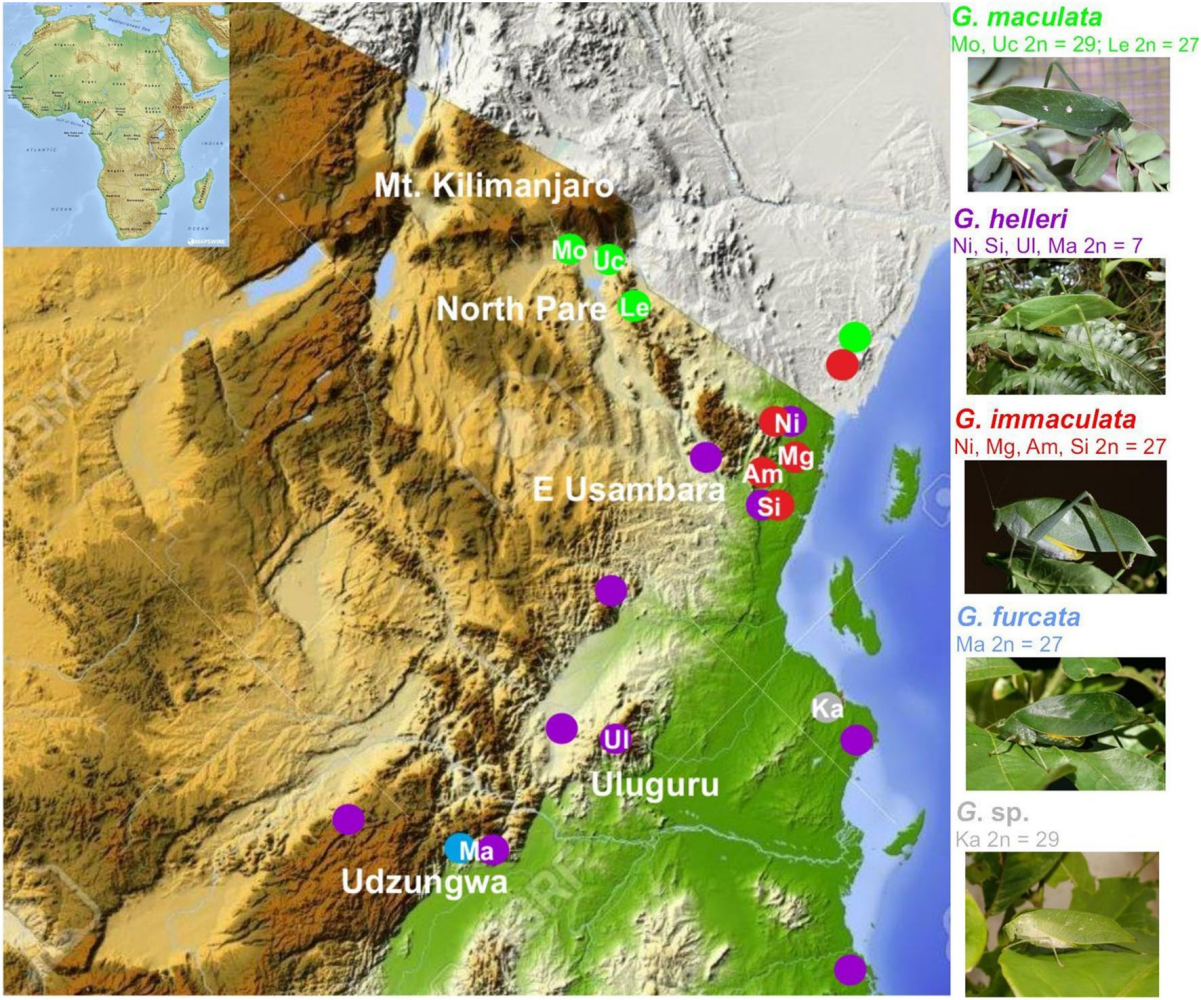
Map of geographical distribution and so far known localities (empty circles) and sampling points for *Gonatoxia* populations in East Africa. Each locality has a color key according to the species name (right side). Photos C. Hemp. Abbreviations in circles are given in Table S1. Diploid chromosome numbers (2n) and localities for species are plotted near the name of the species. The map was made using Google map [https://www.123rf.com/photo_11687795_tanzania-shaded-relief-map-surrounding-territory-greyed-out-colored-according-to-elevation-includes-.html (Projection: Mercator Extents: 28.3/41.5/-12.7/0 Data source: NASA)] and created by E. Warchałowska-Śliwa.

Chromosome diversity of phaneropterine bushcrickets from Eastern Africa shows that most genera are characterized by karyotypic constancy among species, in particular by absence or only a low level of interspecific variability of chromosome numbers. Genomes of three Phaneropterinae genera have uniform karyotypes, with respective chromosome numbers of 2n♂ = 30 + X0 in *Parapyrrhicia* Brunner von Wattenwyl, 1891^63^ and in *Plangia* Stål, 1873^43^, and 2n♂ = 28 + X0 in *Eurycorypha* Stål, 1873^37^. In contrast to this apparent uniformity, an extreme case of rapid chromosome evolution was detected in *Gonatoxia* Karsch, 1889 species^3,46^. *Gonatoxia* is a very fascinating African genus characterized by a high chromosome variability rarely observed in tettigoniids (both in chromosome number and structure, 2n♂ = 28 + X0, 26 + X0 or 6 + X0) and chromosomal polymorphism within and between species. These bushcrickets are fully alate Phaneropterinae, perfectly adapted to the foliage of forests. Only in the last couple of years more information on the bioacoustics^64^ and cytogenetics^3^ of this genus became available, and new species were described^46^. *Gonatoxia maculata* Karsch, 1889 (2n♂ = 28 + X0 and 2n = 26 + X0), a species of savanna woodlands and deciduous dry forests, has the most widespread distribution of the genus, with individuals recorded from Somalia, Kenya and Tanzania while *G. furcata* Hemp, 2016 (2n♂ = 26 + X0) and *G. immaculata* Karsch, 1889 (2n♂ = 26 + X0) are endemics occurring in wet lowland and submontane forests and coastal and lowland wet forests, respectively. *Gonatoxia immaculata* is only known from localities in the East Usambara Mountains and might also occur e.g. in the Nguru Mountains further south, while *G. furcata* is at present known only from the Udzungwa Mountains*. Gonatoxia helleri* Hemp, 2016 (2n♂ = 6 + X0) was found at many localities syntopically with *G. immaculata* and *G. furcata*, overlapping with their ranges (Fig. 1). Also, its ecological niche seems to be broader than those of other species of the genus, occurring from coastal and lowland wet forests (e.g. East Usambara Mountains) up to montane elevations (e.g. Uluguru Mountains). *Gonatoxia helleri* is an especially interesting species because it has the lowest number of chromosomes (2n♂ = 6 + X0) both within its genus and among the Phaneropterinae. Thus, even the most basic characteristics of karyotypes seem to be useful tools to apply in the taxonomy of *Gonatoxia*^3,46^.

The aim of this study was to describe the evolutionary relationships and chromosomal changes that created the karyotypes observed in *Gonatoxia* today. Chromosomal painting was useful for the definition of structural rearrangements, and molecular cytogenetics allowed us to detect homologies between related taxa. We mapped the chromosomal data obtained here, including previous data of *G. helleri*^3^ to test chromosomal evolution in the whole genus. Additionally, we have produced and used a phylogenetic tree based on cytochrome c oxidase subunit I (COI) sequences. The mitochondrial COI gene is unique enough to detect and delimit species of invertebrates^65–69^. The combination of chromosomal and DNA barcode data in comparative phylogenetic studies has been already used to clarify the species position (e.g. Lepidoptera and Diptera^70,71^). This enabled two major hypotheses to be tested: (1) chromosome numbers are correlated with phylogenetic lineages, (2) different types of characters (karyotypes and DNA sequences) exhibit geographical differences related to species distribution.

## Results

### Karyotype analysis

**Figure 2:**
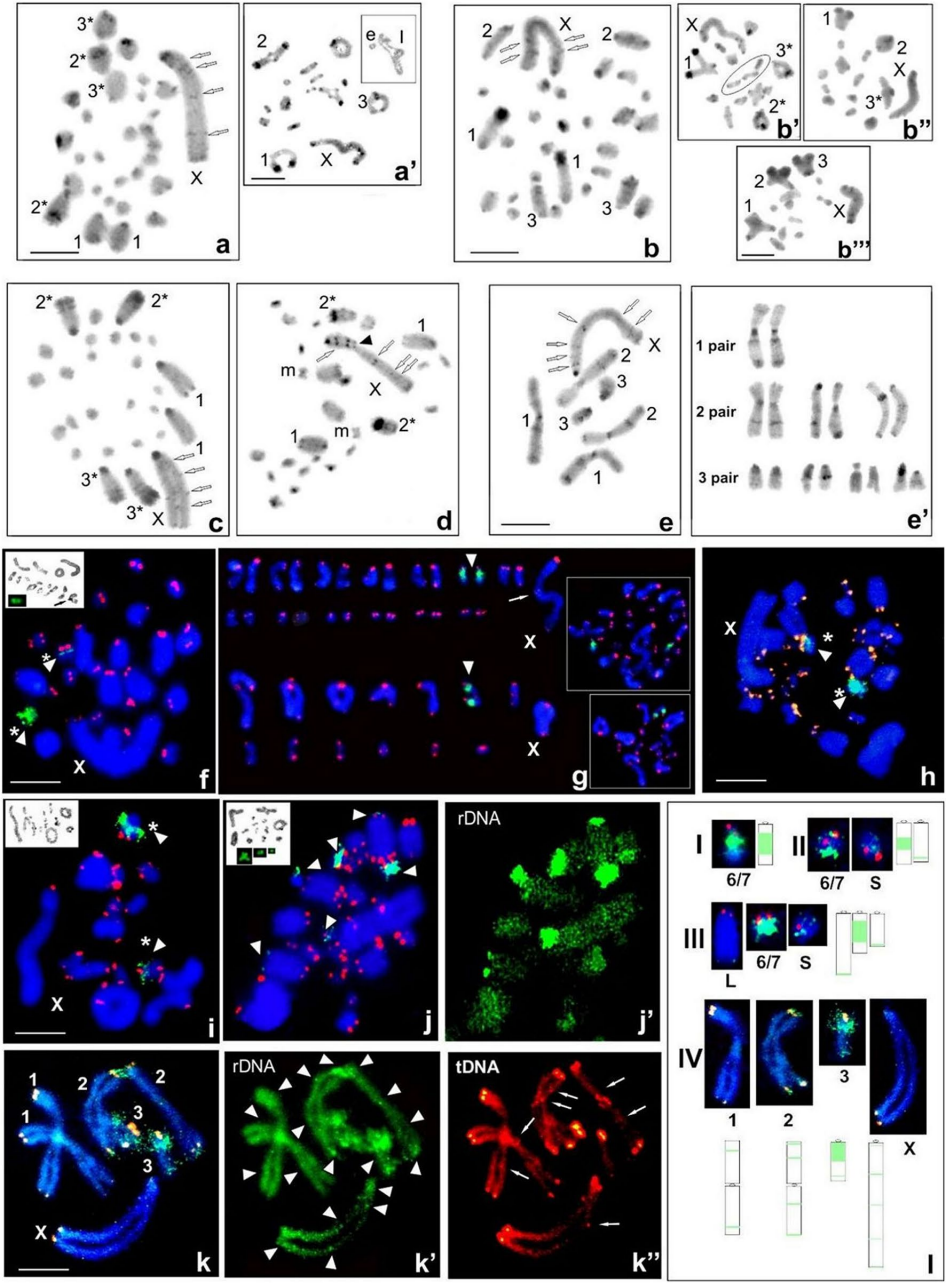
Examples of C-banding in spermatogonial metaphase (**a**, **b**, **c**, **d**, **e**) and diakinesis/metaphase (**a’**, **b’**, **b’’**, **b’’’**), example of fluorochrome-sited heterochromatin **(f**, **j**; in the bottom left corner) and silver staining (**f**, **i**, **j**; in the bottom left corner**)** as well as FISH with both 18S rDNA (green, arrowhead) and telomeric DNA (red) probes in spermatogonial metaphases **(f**, **g**, **h**, **j**, **j’)** and diakinesis **(i)** for the following *Gonatoxia* taxa: *G. maculata*, 2n = 29 (**a**—Uc CH7961, **a’**—Uc CH7963, **f**—Uc CH8043) and 2n = 27 (**g**—Le CH2622), *G.* sp, 2n = 29 (**b**, **b’-b’’’**—CH8046), *G. immaculata* 2n = 27 (**c**—Am CH8499, **i** Mg CH8753, **j**, **j’**—Si CH8622), *G. furcata* (**d**, **h**—Ma CH8047), *G. helleri* (**e**—Si CH8162, **e’**—different individuals, **k**—Ul HE89). Open arrows indicate interstitial C-bands on the X chromosome (**a**, **b**, **c**, **d**, **e**); arrowhead—secondary construction in the X chromosome (**d**); asterisks (*) marked distal C-bands in 2, 3 pairs with heterochromatin heteromorphism (**a**, **b’ b’’**, **c**, **d**); rearrangements such end-to-end association (**b’**) loops (l) and additional elements (**a’** in upper right corner); m—metacentric. White arrowheads point the chromosomal location of rDNA cluster of one (**f**, **g**, **h**), two (**i**), three (**j**, **j’**) pair/bivalent coincides with an active NOR/s (**f**, **i**, **j**—black arrows) and CMA + (**f**, **j**; in the bottom left corner) as well as more clusters rDNA near centromeric, interstitial and telomeric regions of the chromosomes (**k’**). ITS in the X chromosome (**g**) and all chromosomes (**k”**) is marked by white arrows; asterisks (*) marks differences in size between homologous chromosomes (**f**, **h**, **i**). ). (**l**) Scheme summary of the distribution of the 18S rDNA (green) repeat of *G. maculata* (I), *G. immaculata* (II and III), and *G. helleri* (IV); 6/7—chromosome pair, a slash between two numbers indicate imprecise identification of the pair (I, II, III), S—small-sized and L—large-sized autosomes (II, III), 1, 2, 3—the number of chromosome pair, X—sex chromosome (IV). Bar = 10 µm.

A chromosome comparison of five *Gonatoxia* taxa revealed differences between their karyotypes. At the species level and between localities, the variation in the diploid number in males ranged from 2n = 28 + X0 and 26 + X0 to 2n = 6 + X0, in all cases with an X0 sex chromosome system (Table S1). The physical characteristics of the karyotypes, including chromosome number (2n), chromosome morphology, the fundamental number of chromosome arms (FN), locations of 18S rDNA and telomeric DNA clusters, as well as C- and fluorochrome banding patterns (DAPI and CMA_3_) and active nucleolus organizer regions (NORs) including those described previously^3,46^ and in the present study, revealed differences between species as well as between and within populations (Fig. 2).

The standard chromosome complement of *G. maculata* (Mt. Kilimanjaro: localities Moshi, Mo; Uchira, Uc) and *Gonotaxia* sp. (Kazimzumbwi Forest Reserve, Ka) is characterized by 2n = 28 + X0, FN = 29 in the male and 28 + XX, FN = 30 in the female (Fig. 2a,a’,b,f). However, in one male of *G. maculata* collected in the North Pare Mountains (Lembeni, Le) (Fig. 2g) and in all individuals of *G. immaculata* (East Usambara: Nilo, Ni; Sigi, Si; Amani, Am; Magoroto Forest Estate, Mg)*,* the chromosome number was reduced to 2n = 26 + X0/26 + XX, FN = 27/28 (male/female) (Fig. 2c,i,j). In both of the latter species all chromosomes were acrocentric, and the X chromosome was the largest element in the set. It is worth noting that intraspecific variability of the diploid number, caused by instability or mosaic cells, was observed in the same individual based on mitotic (2n = 28 + X0, 27 + X0, 26 + X0, 25 + X0, 24 + X0), and meiotic division [14 + X (Fig. 2b’’), 13 + X (Fig. 2b’’), or 12 + X (Fig. 2b’’’)] in *G. maculata* (Uc: CH7961♂, CH7964♂), *G.* sp. (Ka: CH8046♂) and *G. immaculata* (Ni: CH8244♂) (Table S1—voucher number underlined). Additionally, these specimens and rarely others (*G. maculata* Uc: CH7963♂ and *G.* sp. Ka: CH8552♂), showed structural rearrangements such as end-to-end association (Fig. 2b’), asynapsis, loops or additional elements during meiosis (diakinesis, metaphase I) (Fig. 2a’ in the upper right corner). Individuals of *G. furcata* (Udzungwa Mountains: Mangula Gate, Ma) had a chromosome number of 2n = 26 + X0/26 + XX, FN = 29/30 (male/female); in this karyotype one medium/small-sized pair of chromosomes was metacentric, while others were acrocentric, and the sex chromosome (X) was the largest element in the karyotype (Fig. 2d).

The chromosome complement of *G. helleri* had 2n = 7 (6 + X0), FN = 10–13 in the male, and 2n = 8 (6 + XX), FN = 11–14 in the female. In this karyotype, the first long pair of autosomes was metacentric, whereas the second long and small third pairs were polymorphic in respect to the morphology of homologous chromosomes in specimens from the analyzed localities (Fig. 2e,e’).

### Physical mapping of 18S rDNA and telomeric DNA repeats

As indicated by the data presented in Table S1, independently of the chromosome number, in karyotypes of individuals of *G. maculata*, *G.* sp. and *G. furcata*, FISH revealed a single large cluster of rDNA per haploid genome. This was always located on one medium pair of autosomes (6/7th) in the interstitial region (Fig. 2f,g,h,l). In most samples of *G. immaculata,* two 18S rDNA clusters were evident on the 6/7th bivalent, interstitially located, and on the small bivalent, near the telomeric region (Fig. 2i,l). FISH revealed rDNA on three autosomal pairs (6/7th in the interstitial region and distally located, with low-intensity on short and long autosomal pairs) in only one male of *G. immaculata* (Si: CH8622) (Fig. 2j,l). Sometimes the rDNA-signal varied in size between homologous chromosomes (Fig. 2f,h,i). All examined specimens of *G. helleri* had similar rDNA signals, located in the pericentromeric, interstitial and telomeric regions, usually connected by C-positive regions. The acrocentric/subacrocentric 3rd autosomal pair carried a very large rDNA signal, located near the centromeric region and an interstitial minor 18S rDNA cluster. Additionally, low-intensity clusters of 18S rDNA were observed on the 1st and 2nd pair and the X chromosome, in different chromosome regions (Fig. 2k,k’,l). FISH analysis with the (TTAGG)_*n*_ probe (tDNA-FISH) showed signals at the distal end of each chromosome in the analyzed species. Sometimes the size of clusters on the ends of homologous chromosomes could also vary (Fig. 2f,h.i). A telomeric signal was not observed in the centromeric region of the bi-armed medium sized chromosome pair in *G. furcata* (Fig. 2h). In addition to the typical telomeric signals, interstitial hybridization signals (ITSs) were observed in the middle, near the secondary construction of the X chromosome of *G. maculata* (Fig. 2g). In *G. helleri,* ITSs were observed on the interior regions of all chromosomes (Fig. 2k’’). The position of ITSs seems to agree with a fusion, and/or inversion points of ancient rearrangements. The variation in the intensity of FISH signals was not at random, with a large signal near the centromere in the 2nd and 3rd chromosome pair, depending on the karyomorph, and small signals in the autosomes. Additional weak ITSs were observed at low copy numbers in the subterminal/medial position in the autosomes and in the sex chromosome (Fig. 2k’’).

### Heterochromatin pattern

After both C-staining and fluorochrome DAPI/CMA_3_ double-staining, chromosome regions in *G. maculata, G.* sp., *G. immaculata* and *G. furcata* showed discrete quantitative and qualitative differences between the analyzed specimens, regardless of the species, in terms of the amount of constitutive heterochromatin. All species/specimens, both with 28 + X0 and 26 + X0 chromosomes in the male, had paracentromeric C-bands, which varied in size (Fig. 2a,b’,b’’,c,d). In the long-sized (2nd, 3rd) autosomes and the X chromosome, these C-bands occupied the region next to the centromere (thick C-bands), whereas C-patterns restricted to the centromere (thin C-bands) were seen in most medium and small-sized autosomes.

In the analyzed species, distal thin C-bands were located on most autosomes, with the exception of those present on the 2nd chromosome pair, where a double/thick C-band was clearly seen. Thin paracentromeric C-bands, interstitial and distal/telomeric thin bands were observed in the X chromosome (Fig. 2a,b,c,d,e). The X chromosome carried a secondary construction located near the C-band (Fig. 2d). Generally, thick/thin C-bands were visualized with bright/weakly homogenous DAPI + /CMA_3_ + signals (containing both AT- and GC-rich regions) in the paracentromeric and distal regions. In the analyzed specimens of *G. maculata*, *G*. sp. and *G. furcata*, DAPI- (negative) and weakly stained by CMA_3_ + (GC-rich) signals were localized interstitially in the 6/7th bivalent, and distally in small and long bivalents in *G. immaculata.* These CMA_3_ + bands coincided with one, two or three active NORs and rDNA/FISH signals (Fig. 2f,i,j). In some individuals, heteromorphism of C-bands was observed (indicated with an asterisk in Fig. 2a,b’,b’’,c) in terms of the size on the respective homologous chromosome. The distribution of the constitutive heterochromatin in *G. helleri* is shown in Fig. 2e,e’.

### Molecular phylogenetic analysis

**Figure 3:**
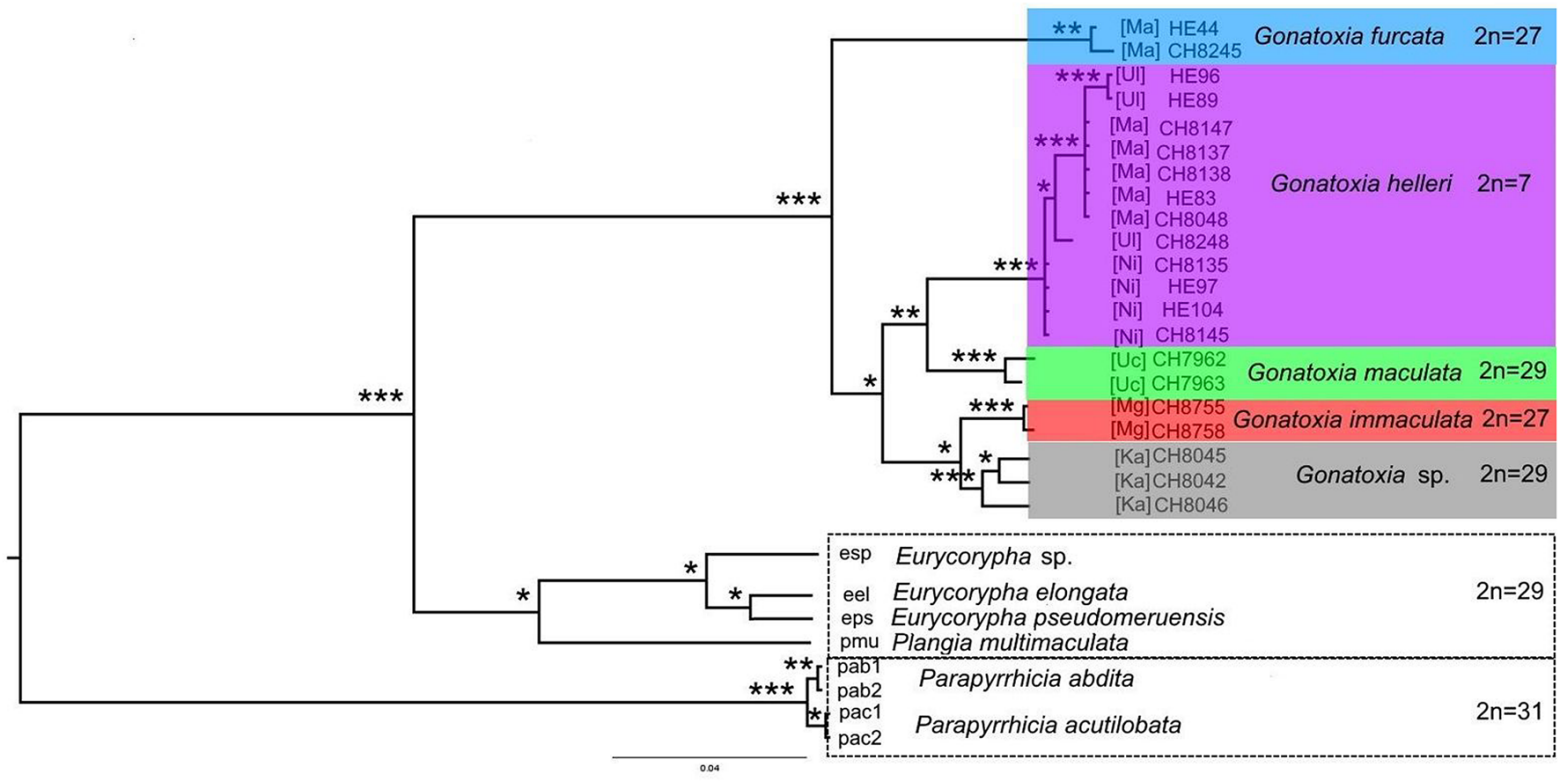
Phylogenetic reconstruction of *Gonatoxia* based on COI sequences with *Eurycorypha, Parapyrrhicia* and *Plangia*. Posterior probability/bootstrap values are indicated by *** = 1/100%, ** = 0.99–0.95/99–95% and * ≤ 0.94/94% (above branches). Diploid chromosome numbers for species are plotted on the tree. Acronyms at the tip labels are meant geographic areas: [Ma]—Udzungwa Mountains, Mangula Gate, [Ul]—Uluguru Mts, [Ni]—Nilo forest reserve, [Uc]—Mt. Kilimanjaro, Uchira, [Mg]—East Usambara Mountains, Magoroto Forest Estate, [Ka]—Kazimzumbwi Forest Reserve. Clades within *Gonatoxia* are indicated by colours corresponding to localities in Fig. 1. Scale bar: number of substitutions per nucleotide position.

Unfortunately, sequencing was not successful for 5 specimens of *Gonatoxia: G. maculata* from Lembeni (Le) (for 1 specimen) and *G. immaculata* from Nilo (Ni) (for 2 specimens), Sigi (Si) (for 1 specimen) and Amani (Am) (for 1 specimen). The aligned COI dataset had a length of 620 bp, with a total of 255 polymorphic characters, of which 237 were parsimony-informative. The model of substitution was the symmetrical gamma distribution model (SYM + G). The obtained BI and ML topologies were congruent (Fig. 3). Both topologies strongly supported the monophyly of *Gonatoxia* and placed it as a sister clade to the genera *Eurycorypha* and *Plangia.* These three genera clustered as sister taxa to species of the genus *Parapyrrhicia*.

Analyzed specimens of *Gonatoxia* clustered analogous to their morphology, comprising 5 species (posterior probability (PP) = 1.00 and bootstrap value (BV) = 100%, Fig. 3). *Gonatoxia furcata* from the Udzungwa Mountains (Mangula Gate, Ma) with the diploid chromosome number (2n) = 26 + X0 was the sister taxon to all other *Gonatoxia* species (PP ≤ 0.94 and BV ≤ 94%). *Gonatoxia immaculata* from the East Usambara Mountains (Magoroto Forest Estate, Mg) with 2n = 26 + X0 was the sister taxon to *G*. sp. from Kazimzumbwi Forest Reserve (Ka) with 2n = 28 + X0 (PP ≤ 0.94 and BV ≤ 94%) and these two were the sister taxa to *G. maculata* from Mt. Kilimanjaro (Uchira, Uc) with 2n = 28 + X0 and the North Pare Mountains and *G. helleri* from various localities (Mangula Gate, Ma; Uluguru Mts, Ul; Nilo forest reserve, Ni) with 2n = 6 + X0 (PP = 0.99–0.95 and BV = 99–95%). Small molecular differences at the population level could be detected between *G. helleri* specimens from the East Usambara, Udzungwa and Uluguru Mountains, despite this they formed one subclade within the *Gonatoxia* group.

**Figure 4:**
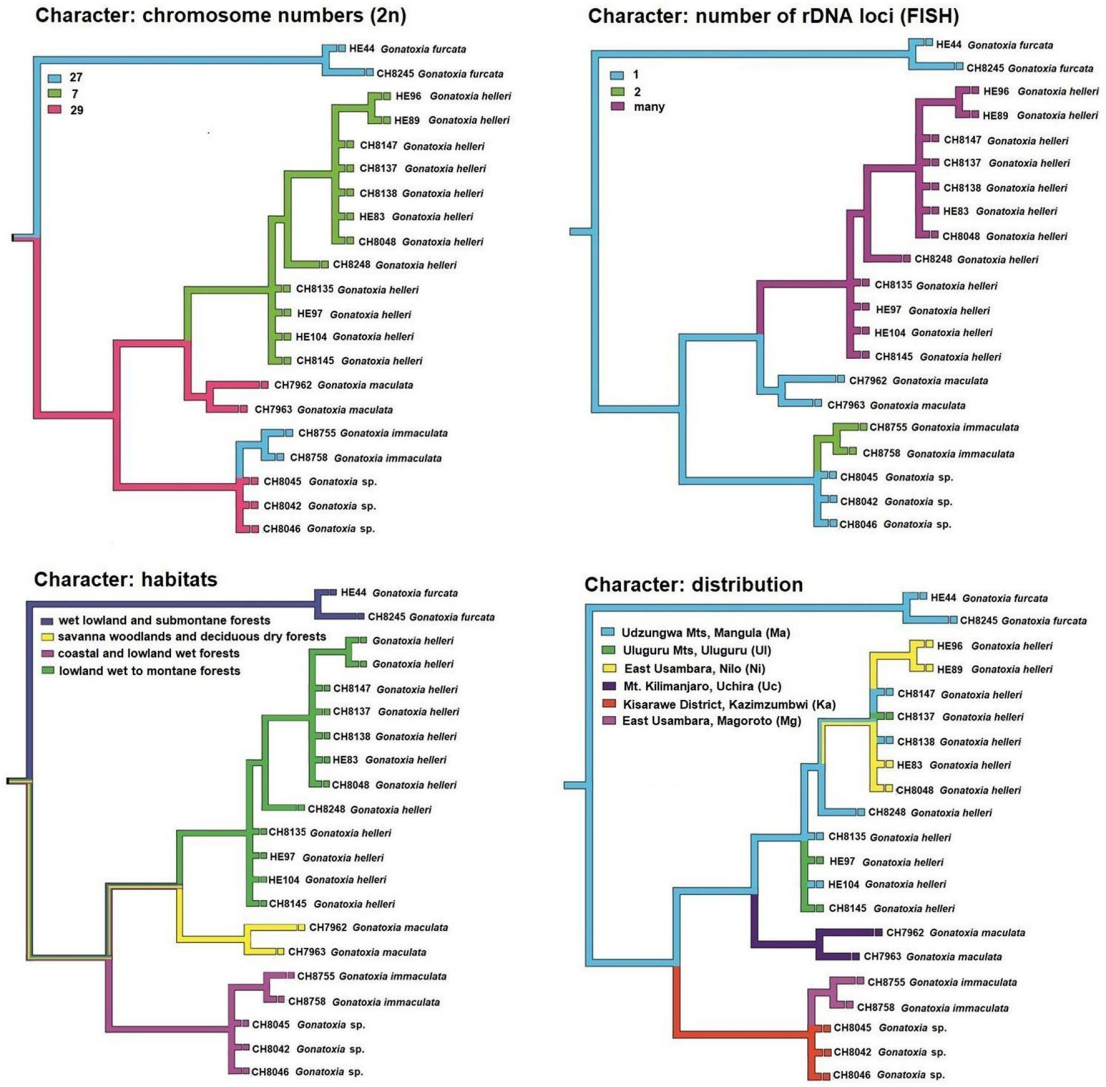
Mapping of chromosome characters, habitats and distribution onto the phylogenetic tree of *Gonatoxia* species. State reconstruction was estimated using the pruned phylogenetic tree topology from Fig. 3.

Mapping the chromosomal characters onto the phylogenetic tree showed three groups with different degrees of polymorphism in chromosome number and the number of FISH-rDNA loci (Fig. 4). However, four and six groups were distinguished comparing habitats and distribution on the COI tree, respectively (Figs. 4, S1) confirming small differences between several specimens of *G. helleri*.

## Discussion

The examination of karyotypes presented in this study provides insights into a hidden variation in *Gonatoxia,* on a population as well as the species level. We wanted to examine the mechanisms of karyotype evolution in a phylogenetic context. This study presents the preliminary results of molecular investigations, in an attempt to discover species boundaries in the chromosomally diverse taxa of the genus *Gonatoxia.*

### Chromosome evolution and diversification

Our cytogenetic examination confirms previous study findings^3,46^ and shows that *Gonatoxia* exhibits a high chromosome differentiation between species and complicated cytogenetic systems (polymorphism), which are unusual in tettigoniids. Despite this, differences between karyotypes of populations in the same species/taxa and between species may be concealed by the distribution of specific chromosomal markers, the extensively investigated major rDNA genes. The observed diversity of chromosome numbers in this genus could theoretically be the result of chromosomal rearrangements (CRs) emerging from the ancestral/model karyotype (2n = 30 + X0) found in most tettigoniids (e.g.^71^) and most other African Phaneropterinae genera^40–42,63^. However, this model chromosome number was not found in *Gonatoxia*. For the karyotype 2n = 28 + X0 of *G. maculata* (Mt. Kilimanjaro: Mo, Uc) and *G.* sp. (Kazimzumbwi Forest Reserve, Ka), it is hypothesized that the autosome number was reduced by one tandem fusion. This number of chromosomes, including only acrocentric chromosomes, has been also reported for African species of *Eurycorypha* and *Plangia*^37,43^. However, the same number is also found in more distantly related genera^72^, indicating several independent reductions in this subfamily. On the other hand, a reduced chromosome number of 2n = 26 + X0, probably caused by two fusions (all chromosomes being acrocentric) in one specimen of *G. maculata* from Lembeni (North Pare Mountains) and *G. immaculata* from all localities (East Usambara Mountains: Ni, Si, Am, Mg)*,* might imply that a chromosomal mutation happened probably independently in individuals. The chromosome number variation between the populations of *G. maculata* (2n♂ = 29, 27), which most likely are different chromosomal races, suggests ongoing differentiation in isolated populations (Table S1). In tettigoniids, chromosomal races have previously been described in the *Steropleurus martorelli* complex (Bradyporinae), a coastal species endemic to the Iberian Peninsula with diploid chromosome numbers in the male ranging from 29 to 23^73^. In *G. furcata* (2n = 27) from the Udzungwa Mountains, however, a different mechanism in reducing the chromosome number must be assumed. This reduction was probably caused by one fusion and an additional Robertsonian translocation (i.e. fusion-fission cycle) between two small pairs of autosomes. Besides these changes in chromosome number, it should be noted that in some individuals of *G. maculata*, *G*. sp. and *G. immaculata,* variation of the chromosome number in the same individual (instability/mosaic cells) in mitotic division, and irregularities in meiotic cell division, such as translocation, asynapsis, loops or additional elements, were observed—although rarely. As a consequence of these mechanisms, an aneuploid-dysploid mosaicism of the chromosome complement cannot be excluded. Diploid chromosome rearrangements (pseudoaneuploidy) are often assumed to be the dominant mechanism of a basic chromosome number reduction in grasses (e.g.^74^). In turn, the main characteristics of the karyotype of *G. helleri,* with only seven large chromosomes (2n = 6 + X0), are a reduced chromosome number and the asymmetrical karyotype (karyomorphs) rarely being found in tettigoniids. Additionally, in this species irregularities in the course of meiosis compared to other *Gonatoxia* species were observed. This reflects the derivation by multiple rearrangements of the ancestral karyotype, probably by Robertsonian translocations and/or tandem fusions and inversions. Additionally, in some individuals, a diploid number was observed with intra-individual variability among cells, with 14 (male) and 16 (female) pairs of chromosomes, probably corresponding to tetraploid levels^3^. In this species, an incompatible meiotic configuration and a level of heterozygosity in some populations may have been a precondition of colonizing new habitats and might be a case of adaptive diversification in *G. helleri*^3^.

Our cytogenetic data show that the East African genus *Gonatoxia* exhibits a great variation in its karyotypic macrostructure e.g. the number of chromosomes and the morphology and the distribution of specific chromosomal markers, such as in the extensively investigated tettigoniid major rRNA genes (18S), telomeric DNA and constitutive heterochromatin. The application of different staining techniques (both classical and molecular) provides useful markers for the characterization of the chromosomes of phaneropterines and other tettigonids^29–32^, and is important for the development of genus or species-specific models of karyotypes. The karyotypes of individuals of *G. maculata*, *G.* sp*.* and *G. furcata* (regardless of the number of chromosomes), have a single 18S rDNA/active NOR-bearing chromosome pair, which is typical of most African and European phaneropterid taxa examined so far. Generally, rDNA loci in Phaneropterinae were situated in the pericentromeric region, which would imply that this chromosomal feature may be plesiomorphic, or is found rarely in the interstitial region (e.g.^29–32^). Individuals of *G. maculata*, *G.* sp*.* and *G. furcata*, similar detected in one *Parapyrrhicia*^63^ and many *Eurycorypha* species^37^, have 18S rDNA clusters placed in an interstitial position, which may have resulted from CRs (peri- or paracentromerc inversions, tandem fusions), transposable element insertions or ectopic recombinations^75^. In contrast, *G. immaculata* shows inter- and intraspecific polymorphism including the number (two or rarely three) and position (interstitial—similar to other species on 6/7th pair and distal on one or two small pairs) of 18S rDNA sites. Two pairs of chromosomes bearing DNA clusters appear to be potentially useful cytogenetic markers for distinguishing this species among other *Gonatoxia* species with 29/27 chromosomes in their karyotypes. Interestingly, the occurrence of three DNA clusters in *G. immaculata* was connected with intraspecific variation. This genetic variance and instability may be the result of rapid recent and/or ongoing diversification and/or frequent hybridization. The diversity of the number and distribution of 18S rDNA between *Gonatoxia* species may be explained by (i) ectopic recombination, as described for the scorpion *Tityus obscurus*^45^; (ii) translocations, considering that heterochromatic regions rich in rDNA are also considered hotspots for chromosome breaks^76^; or (iii) transposition of active transposable elements^77,78^.

Generally, our results demonstrate a coincidence of the locations of rDNA loci and an active NOR/s, as well as C-positive and G–C rich heterochromatin regions, similar to other African phaneropterids e.g.^31,63^). However, compared to phaneropterines and other tettigoniids, the chromosomal distribution of major 18S rDNA signals in *G. helleri* is unique. Specifically, there are differences in the location (pericentromerically, interstitially and distally), a high number of sites with differing intensity of hybridization signals, and the presence of interstitial telomeric sequences (ITSs) near the pericentromeric and interstitial regions and/or near the telomeric region. The presence of several sites with ITSs (identified in addition to the terminal telomeric sequences) in *G. helleri*^3^, and a single ITS near the secondary construction in the X chromosome of *G. maculata*, confirm the hypothesis that this karyotype/these karyomorphs could be the result of telomere-telomere fusions of the chromosomes, inversions (intra-chromosomal rearrangements), unequal crossing over, or the insertion of telomeric DNA into unstable sites during the repair of double-strand breaks^79^. ITSs have been regarded as remnants of chromosomal fusions promoted by repetitive subtelomeric elements as well as inversions^80,81^.

### Phylogenetic relationships

In our phylogenetic reconstruction, the five analyzed *Gonatoxia* species form a monophyletic group. *Gonatoxia furcata*, endemic to the Udzungwa Mountains (Ma), clusters at the base of *Gonatoxia,* being the sister group to all other species of this genus. *Gonatoxia immaculata* from the East Usambara Mountains (Mg) and *Gonatoxia* sp. from Kazimzumbwi Forest Reserve (Ka) further south at the coast formed one sister clade. *Gonatoxia maculata*, probably a widespread species in East Africa, and *G. helleri* (Ul, Ma, Ni), another widespread species, formed the sister clade. Observed chromosome numbers agree well with the molecular information gained from the COI gene and their habitats as well as their distribution. *Gonatoxia furcata*, a rare species only found in low numbers in the Udzungwa Mountains, could have a reduced chromosome set due to habitat constriction, individuals of this species having a small area of occurrence. Local adaptation could explain the evolution of many chromosome fusions, which are some of the most common chromosome rearrangements in nature^82^. This mechanism of chromosome reduction, in connection with a restricted habitat, has already been discussed for the hexacentrine genus *Aerotegmina*^38^, which includes species with a mix of an ancestral number (2n = 32 + X0) and a reduced number of chromosomes (2n = 26 + X0). Similar events have been suggested to have affected the speciation of African mole-rats (Rodentia) of the genera *Cryptomys* and *Fukomys,* endemic to sub-Saharan regions^83^, or *Arvicanthis*^84^, which are characterized by extreme chromosomal variation.

*Gonatoxia furcata* with a reduced chromosome number (2n = 26 + X0) exhibits the ancestral (plesiomorphic) condition, whereas most other taxa had a higher number of chromosomes (2n = 28 + X0). Only *G. helleri*, probably the most recently evolved species, with a wide distribution and occupying a wide ecological niche from lowland to montane forests, had an extremely reduced chromosome number. Additionally, small molecular differences were detected between several specimens of *G. helleri* from different localities and habitats (lowland wet forest in the East Usambaras to lowland wet forest in the Udzungwas and montane forest in the Ulugurus). Specimens from the Udzungwa and Uluguru Mountains clustered together, showing the geographical proximity of these mountain ranges, while specimens from the further north located East Usambara Mountains formed a separate cluster at the base of the clade of *G. helleri*. These clusters of *G. helleri* also suggest that populations are isolated from each other and that speciation is currently in progress in this species.

## Conclusion

Our integrative approach, combining the cytogenetic and phylogenetic methods presented herein, constitutes a step towards a better understanding of chromosomal organization and relationships within *Gonatoxia* and other African Phaneropterinae. These data showed to be convincing tools, supporting systematic and taxonomic research and uncovering hidden biodiversity. Our results show that *Gonatoxia* represents a genetically diverse group, showing variation in terms of the number and morphology of chromosomes and the distribution of DNA clusters. Considering the current geographical range, the isolation of populations of one species and their respective karyotypes, differences in rDNA/NOR numbers (*G. immaculata*, *G. helleri*), and the abundance of chromosomal heterozygotes (*G. helleri*), this probably represents a case of preconditioning to successfully colonize new habitats and to cope with changing environments. The fixation and/or heteromorphism of different CRs, by geographic isolation and genetic drift could have been triggered by climatic events that changed the environment, leading CRs to play some role in lineage diversification. The observed chromosome numbers and their distribution agree well with the molecular information gained from the COI gene. The knowledge compiled here provided useful information for understanding the speciation processes and faunal formation of African insects. Thus, we here presented data on the genus *Gonatoxia* serving as a model to understand biogeographic patterns in East Africa through a combination of analysing chromosome numbers and structures, and combining them with molecular methods and information on the habitat.

## Material and methods

### Taxon sampling

From 2014 to 2019, samples of 66 specimens were analyzed from male adults or nymphs and molted females or nymphs, belonging to five species of *Gonatoxia* (14 individuals, 3 locations—*Gonatoxia maculata*; 5 individuals, 1 location—*G.* sp.; 12 individuals, 4 locations—*G. immaculata*; 2 individuals, 1 location—*G. furcata*; and 33 individuals, 4 locations—*G. helleri*). The sampled bushcrickets were taken in the field from 10 populations in northern Tanzania (Table S1; Fig. 1).

### Cytogenetic procedures

Testes, ovaries, and somatic hepatic caeca were dissected, incubated in hypotonic solution (0.9% sodium citrate), fixed in ethanol—acetic acid (3:1, *v/v*), and squashed in 45% acetic acid. Cover slips were removed using a dry ice procedure and the slides were air dried. To determine the distribution of heterochromatin Giemsa C-banding was carried out using the methods described in^85^, the GC/AT-rich chromosomal segments were stained with fluorochromes CMA_3_ (chromomycin A_3_) and DAPI (4’,6-diamidino-2-phenylindole)^86^. The silver staining method (AgNO_3_) for localization of NORs was performed as previously reported^87^. FISH experiments were performed following the protocol of^88^. The rDNA gene was amplified and labeled using primers 18S forward and 18S reverse^89^, and biotin-16-dUTP (Roche Diagnostics GmbH, Germany) by polymerase chain reaction (PCR). Telomere probes were PCR-amplified and labeled using the primers TTAGG_F and TTAGG_R^89^ and digoxigenin-11-dUTP (Roche Diagnostics GmbH, Germany). FISH signals were detected using avidin-FITC (Invitrogen, USA) and anti-digoxigenin rhodamine (Roche Diagnostics GmbH, Germany). Preparations were counterstained with ProLong Gold antifade reagent containing DAPI (Invitrogen, USA). Images were captured using a Nikon Eclipse 400 fluorescence microscope equipped with a CCD DS-U1 camera and the NIS-Elements BR 3.0 software package. Information was based on analysis of mitotic metaphase (oogonial/spermatogonial) and/or meiotic divisions using both rDNA and telomeric DNA (at least 25 images per individual). In each species/individual, karyograms were reconstructed by arranging homologous chromosomes in decreasing size.

### DNA extraction, amplification, and sequencing

Genomic DNA extraction was carried out from 34 single specimens (including 5 specimens for which sequencing was not successful; 1–12 specimens per *Gonatoxia* species and 1–2 specimens per outgroup species; 1–5 specimens of *Gonatoxia* per location; Table S1), using a NucleoSpin Tissue kit (Macherey–Nagel, Germany) following the manufacturer’s protocol. The mitochondrial partial COI gene was amplified with the primers LCO and HCO^90^. The polymerase chain reaction (PCR) was performed in 20 µl of reaction volume consisting of 2 µl reaction buffer, 25 mM MgCl_2_, 10 mM dNTP mixture, 10 µM forward and reverse primers, 1–3 µl of genomic DNA, 5 U of Gold Taq DNA polymerase (Syngen, Poland), and sterile deionized water. To amplify COI, the following PCR protocol was used: 36 cycles at 94 °C for 1 min, 48 °C for 1 min and 72 °C for 2 min, with the final extension at 72 °C for 7 min. The PCR products were purified with EPPiC Fast (A&A Biotechnology, Poland, following the standard protocol). Sequencing reactions were carried out in 10 µl reactions containing: 1.5 µl of sequencing buffer, 1 µl of the BrilliantDye v3.1 Terminator Cycle Sequencing Kit (Nimagen, Nijmegen, The Netherlands), 1 µl of primer (forward or reverse), 3.0 µl of the purified products and 3.5 µl of sterile water. The sequencing reaction was as follows: 3 min at 94 °C, 25 cycles of 10 s at 96 °C, 5 s at 55 °C and 90 s at 60 °C. The 29 individuals which gave positive results on PCR amplification of the COI gene of *Gonatoxia, Eurycorypha, Plangia* and *Parapyrrhicia* were successfully sequenced. The nucleotide sequences of the analyzed specimens were deposited in GenBank under the accession numbers provided in Table S1.

### Sequence alignment and phylogenetic analysis

The obtained nucleotide sequences were edited, assembled into contigs and aligned in CodonCode Aligner v. 8.0 (CodonCode Corporation; https://www.codoncode.com/aligner, March 08, 2018) with default parameters. Sequences were checked for protein-coding frame shifts to detect pseudogenes using MEGA v. X^91^, and compared with sequences from GenBank through a BLAST search. Phylogenetic trees were estimated using Bayesian Inference (BI) and maximum likelihood (ML). The substitution model of evolution was estimated in MrModeltest v. 2 software^92^ using the Akaike Information Criterion (AIC). BI was implemented in MrBayes v. 3.2^93^ with four independent runs, each having one cold and three heated Markov chains. The analysis was run for 6 million generations, with trees sampled every 100 generations. The first 25% of each run was discarded as burn-in. Convergence among the runs was assessed using Tracer v. 1.7^94^. The ML analysis was conducted in IQ-TREE v. 1^95^. Nodal support was assessed using a nonparametric bootstrap with 1000 replicates. To visualize the patterns of evolution in *Gonatoxia*, chromosomal characters, habitats and distribution were mapped according to parsimony criteria in a pruned phylogenetic tree from Fig. 3 using the software Mesquite v. 3.61^96^. To project a tree onto a geographic map we used “phytools” package v. 0.7-70^97^ in R statistical software v. 4.0.3^98^.

## Supplementary Information

Supplementary Information.
