# Supplementary material for: Highly divergent karyotypes and barcoding of the East African genus Gonatoxia Karsch (Orthoptera: Phaneropterinae)
Source: Sci Rep. 2021 Nov 23;11:22781. doi: 10.1038/s41598-021-02110-8 (PMC8610994; doi:10.1038/s41598-021-02110-8)
Supplement: Supplementary file 1 — Supplementary Information. [file 41598_2021_2110_MOESM1_ESM.pdf]

Supplementary Materials

Highly divergent karyotypes and barcoding of the East African genus *Gonatoxia* Karsch (Orthoptera: Phaneropterinae)

Elżbieta Warchałowska-Śliwa[1], Beata Grzywacz[1*], Maciej Kociński[1], Anna Maryańska-Nadachowska[1], Klaus-Gerhard Heller[2], Claudia Hemp[3]

[1]Institute of Systematics and Evolution of Animals, Polish Academy of Sciences, Sławkowska 17, 31-016 Kraków, Poland
[2] Independent researcher, Magdeburg, Germany
[3] ~~University of Bayreuth, Dept. Plant Systematics~~, Bayreuth, Germany

[*]Corresponding author: Beata Grzywacz
Institute of Systematics and Evolution of Animals, Polish Academy of Sciences, Sławkowska 17, 31-016 Krakow, Poland, e-mail: grzywacz@isez.pan.krakow.pl

Table of contents

Table S1. Species of *Gonatoxia*: where and when collected in northern Tanzania, chromosome number and molecular analysis; mosaic individual; *only DNA.

Figure S1. Phylogenetic tree overlaid on the geographic locations of sampled individuals. Vouchers at the tip labels indicate *Gonatoxia* species: [HE44, CH8245] – *G. furcata*, [CH8048, CH8135, CH8137, CH8138, CH8145, CH8147, CH8248, HE83, HE89, HE96, HE97, HE104] – *G. helleri*, [CH7962, CH7963] – *G. maculata*, [CH8042, CH8045, CH8046] – *Gonatoxia* sp., [CH8755, CH8758] – *G. immaculata*. Acronyms on the map indicate geographic areas: [Ma] – Mangula, Udzungwa Mts; [Ul] – Uluguru Mts; [Ka] – Kazimzumbwi, East Usambara; [Mg] – Magoroto, East Usambara; [Ni] – Nilo, East Usambara; [Uc] – Uchira, Mt. Kilimanjaro. The map was created in "phytools" R package version 0.7-80 (https://cran.r-project.org/web/packages/phytools/index.html?fbclid=IwAR2PGOcMtucCVbdPHeoeDE0WmZWxucx5uzFnKByvooDLZris_Q_EMTSyI0g) [98].

Table S1. Species of *Gonatoxia*: where and when collected in northern Tanzania, chromosome number and molecular analysis; mosaic individual; *only DNA.

**Species**	**Localities collected, date and voucher number**	**Geographical coordinates**	**2n (male)**	**FISH rDNA [total]**	**COI voucher/isolate voucher**	**GenBank acc. no.**
Gonatoxia maculata	Mt. Kilimanjaro, southern slopes, Rau forest near **Moshi [Mo]**, lowland wet forest, 800 m, 12.2014; male CH7893; 02.2015; HE16	S 3° 21' 0"<br>E 37° 20' 0"	29	HE16<br>[1]	-	-
	Mt. Kilimanjaro, southern slopes, **Uchira [Uc]**, savanna woodlands, 10. 2015; males: CH7961, CH7963, CH7964, CH7966, CH8043, CH8044, CH8083, CH8084, CH8085; females: CH7962, CH7965	S 3° 25' 0"<br>E 37° 29' 0"	29	CH7961<br>CH7966<br>CH8043<br>CH8044<br>CH8083<br>[1]	CH7962/gma1<br>CH7963/gma2	MW792453<br>MW792454
	Eastern Arc Mts, North Pare Mts, **Lembeni [Le]**, dry deciduous forest, 03.2013; male CH7622	S 3° 47' 0"<br>E 37° 37' 0"	27	CH7622<br>[1]	-	-
***Gonatoxia* sp.**	Pwani Region, Kisarawe District, **Kazimzumbwi Forest Reserve [Ka]**, 150 m, 07.2015; males: CH8046, CH8552; females: CH8042, CH8045, CH8553	S 6° 58' 0"<br>E 39° 3' 0"	29	CH8042<br>CH8552<br>[1]	CH8042/gim3<br>CH8045/gim1<br>CH8046/gim2	MW792452<br>MW792450<br>MW792451
Gonatoxia immaculata	Eastern Arc Mts, East Usambara, **Nilo [Ni]** forest reserve, lowland wet forest, 07-08.2016; males: CH8244, CH8288	S 4° 55' 8"<br>E 38° 39' 42"	27	CH8288<br>[2]	-	-
	Eastern Arc Mts, **Sigi [Si],** 11.2018; CH8622	S 5°06′00″<br>E 38°39′00″	27	CH8622<br>[3]	-	-
	Eastern Arc Mts, East Usambara, **Amani [Am]**, 11.2017; male CH8499	S 5°06'00.8"<br>E 38°37'42.0"		CH8499<br>[2]	-	-
	Eastern Arc Mts, East Usambara, **Magoroto Forest Estate [Mg]**, , 07.2019; males CH8751, CH8752, CH8753, CH8754; females CH 8755, CH8757, CH8758, CH8759	S 5°06'39.0"<br>E 38°45'13.7"		CH8751<br>CH8752<br>CH8753<br>CH8754<br>[2]	CH8755/gim4<br>CH8758/gim5	MW792460<br>MW792461
Gonatoxia furcata	Eastern Arc Mts, Morogoro District, Udzungwa Mts, National Park Headquarters, **Mangula Gate [Ma]**, 300 m, lowland wet	S 7° 50' 6"<br>E 36° 53' 59"	27	CH8047<br>[1]	HE44/gfu1<br>CH8245/gfu2	MW792436<br>MW792437

	forest, 07.2015, 07.2016; male CH8047, female CH8245					
Gonatoxia helleri	Eastern Arc Mts, Morogoro District, Udzungwa Mts, National Park Headquarters, **Mangula Gate [Ma]**, 300 m, lowland wet forest, 01-03.2015; males: CH7949, CH8048, CH8087, CH8088, CH8089, CH8144, CH8145, CH8247; females: CH8072, CH8073, CH 8137, CH8138, CH8139, CH8146, CH8147; 07.2016; male CH8247	S 7° 50' 6" E 36° 53' 59"	7	CH7949 CH8048 CH8088 [many]	CH8048/ghe4 CH8138/ghe16 CH8147/ghe12 CH8137*/ghe15 HE83*/ghe20	MW792447 MW792442 MW792439 MW792441 MW792445
	Eastern Arc Mts, Morogoro District, **Uluguru Mts [Ul]**, forest above Morningside, 02.2016, 03.2016, 07.2016; males: HE89, HE96, HE105, CH8246, CH8251, CH8252, CH8253; females: CH8250, CH8289	S 6°54'19.5" E 37°40'23.8"		HE89 HE96 CH8252 [many]	HE89/ghe2 HE96/ghe19 CH8248*/ghe6	MW792444 MW792443 MW792448
	Eastern Arc Mts, East Usambara, **Nilo [Ni]** forest reserve, lowland wet forest; 12.2015; male CH8134, HE 97, HE104, female CH8135; 02.2016; males: HE97, HE104	S 4° 55' 8" E 38° 39' 42"		HE97 [many]	CH8135/ghe1 CH8145/ghe8 HE97/ghe14 HE104/ghe21	MW792438 MW792449 MW792440 MW792446
	Eastern Arc Mts, East Usambara**, Sigi [Si],** 11.2015; female CH8136; 11.2018: male CH8162	S 5°06′00″ E 38°39′00″		CH8162 [many]	-	-
Eurycorypha elongata	Eastern Arc Mts, East Usambara, Nilo forest reserve, lowland wet forest; 12.2015; male	S 4° 55' 8" E 38° 39' 42"	29	-	HE64/eel	MW792433
Eurycorypha pseudomeruensis	Tabora; 02.2016; male	S 5° 05′ 25″ E 32° 49′ 47″	29	-	HE100/eps	MW792434
***Eurycorypha* sp.**	Eastern Arc Mts, Udzungwa Mts; 07.2015; male	S 7° 46′ 01″ E 36° 49′ 02″	29	-	CH8153/esp	MW792435
Plangia multimaculata	Mwanga; 02.2015; male	S 3° 39′ 32″ E 37° 35′ 55″	29	-	HE30/pmu	MW792459
Parapyrrhicia abdita	Eastern Arc Mts, Udzungwa Mts; 01-02.2016; males	S 7° 46′ 01″ E 36° 49′ 02″	31	-	CH76/pab1 CH84/pab2	MW792455 MW792456
Parapyrrhicia acutilobata	Kisarawe; 02.2015, 07.2015; male, female	S 6° 54′ 56″ E 39° 03′ 35″	31	-	CH30/pac1 CH8049/pac2	MW792457 MW792458

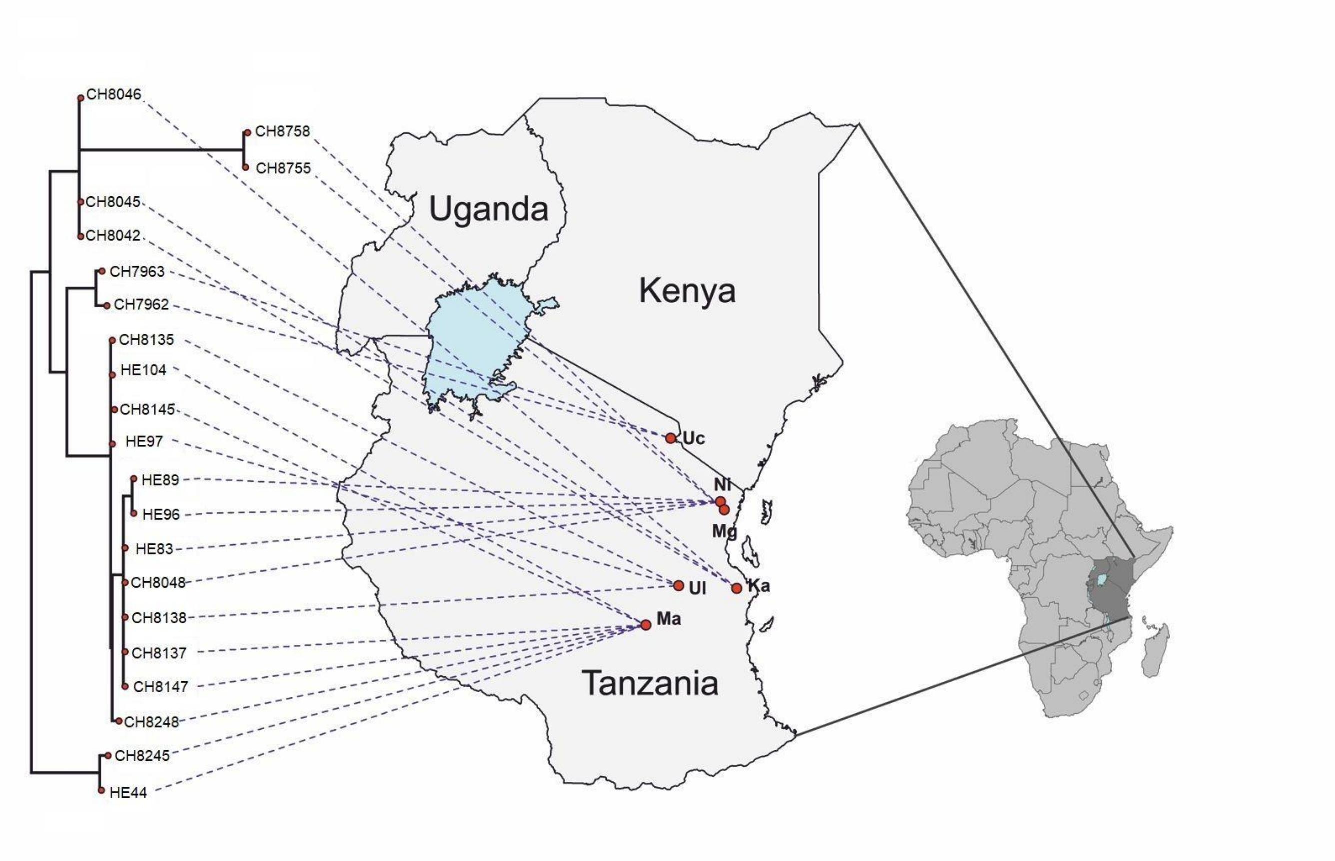

Figure S1. Phylogenetic tree overlaid on the geographic locations of sampled individuals. Vouchers at the tip labels indicate *Gonatoxia* species: [HE44, CH8245] – *G. furcata*, [CH8048, CH8135, CH8137, CH8138, CH8145, CH8147, CH8248, HE83, HE89, HE96, HE97, HE104] – *G. helleri*, [CH7962, CH7963] – *G. maculata*, [CH8042, CH8045, CH8046] – *Gonatoxia* sp., [CH8755, CH8758] – *G. immaculata*. Acronyms on the map indicate geographic areas: [Ma] – Mangula, Udzungwa Mts; [Ul] – Uluguru Mts; [Ka] – Kazimzumbwi, East Usambara; [Mg] – Magoroto, East Usambara; [Ni] – Nilo, East Usambara; [Uc] – Uchira, Mt. Kilimanjaro. The map was created in "phytools" R package version 0.7-80 (https://cran.r-project.org/web/packages/phytools/index.html?fbclid=IwAR2PGOcMtucCVbdPHeoeDE0WmZWxucx5uzFnKByvooDLZris_Q_EMTSyI0g) [98].
